# What is the speed limit of martensitic transformations?

**DOI:** 10.1080/14686996.2022.2128870

**Published:** 2022-10-06

**Authors:** Stefan Schwabe, Klara Lünser, Daniel Schmidt, Kornelius Nielsch, Peter Gaal, Sebastian Fähler

**Affiliations:** a Leibniz IFW Dresden, Institute for Metallic Materials, Dresden, Germany; b TU Dresden, Institute of Materials Science, Dresden, Germany; c Helmholtz-Zentrum Dresden-Rossendorf, Institute of Ion Beam Physics and Materials Research, Dresden, Germany; d Department Application Science, Leibniz-Institut für Kristallzüchtung (IKZ), Berlin, Germany; e TXproducts UG, Hamburg, Germany

**Keywords:** Martensitic phase transitions, time-resolved synchrotron diffraction, shape memory alloys, magnetocaloric refrigeration, thermomagnetic energy harvesting

## Abstract

Structural martensitic transformations enable various applications, which range from high stroke actuation and sensing to energy efficient magnetocaloric refrigeration and thermomagnetic energy harvesting. All these emerging applications benefit from a fast transformation, but up to now their speed limit has not been explored. Here, we demonstrate that a thermoelastic martensite to austenite transformation can be completed within 10 ns. We heat epitaxial Ni-Mn-Ga films with a nanosecond laser pulse and use synchrotron diffraction to probe the influence of initial temperature and overheating on transformation rate and ratio. We demonstrate that an increase in thermal energy drives this transformation faster. Though the observed speed limit of 2.5 × 10^27^ (Js)^1^ per unit cell leaves plenty of room for further acceleration of applications, our analysis reveals that the practical limit will be the energy required for switching. Thus, martensitic transformations obey similar speed limits as in microelectronics, as expressed by the Margolus – Levitin theorem.

## GRAPHICAL ABSTRACT

**Figure:**
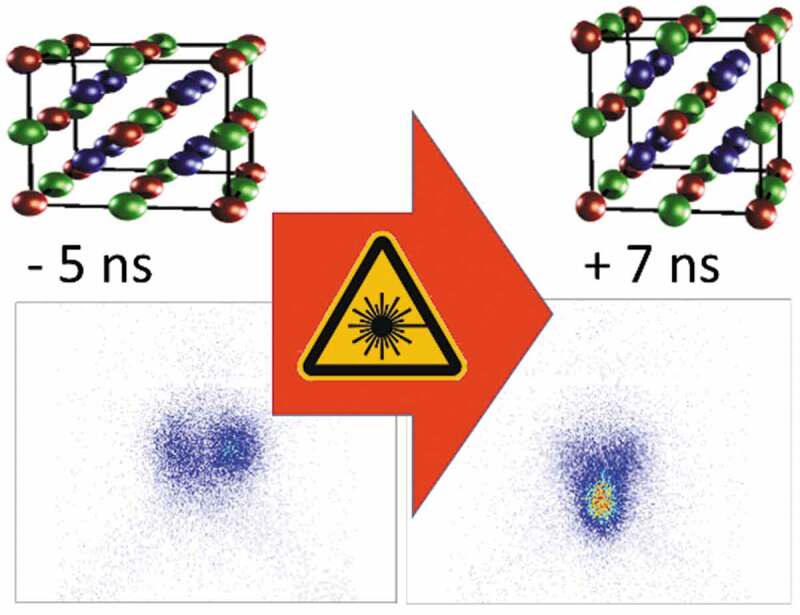

## 1. Introduction

‘Can you make it faster?’ is a question frequently heard by scientists. However, often physical limitations represent an insurmountable speed limit even for the best scientists. A famous example is microelectronics, where the clock frequency of computers had already stopped its exponential increase two decades ago [1]. The physical limit in this case is the energy required for switching between two states, a speed limit predicted some time before it was reached [2]. Thus, it is worth to ask the question of the ultimate shortest timescale already when new functional materials and applications emerge. We focus on martensitic transformations, which switch the material’s crystal symmetry within the solid state by temperature. These transformations are the underlying mechanism for the following emerging applications that will benefit from an increased speed since they use highly reversible cycles achievable only in so-called thermoelastic martensitic transitions. Actuation by shape memory effects is ultimately limited by the speed of this transformation [3]. The same limitation also holds for the reverse process used for sensing [4]. Martensitic transformations occur in the most promising magnetocaloric materials as well, where a higher switching frequency is required to increase the cooling power [5]. The reverse process, which is used to harvest low-grade waste heat by thermomagnetic materials, also benefits from an increased frequency [6]. Here, we experimentally approach the time limit required for a thermoelastic martensitic transformation – well before this speed limit will be reached in these applications.

What is a martensitic transformation and what is known about its speed? A martensitic transformation switches the crystal structure between high symmetry at high temperatures and low symmetry at low temperatures. It is a transformation without diffusion processes between a high-temperature phase called ‘austenite’ and a low-temperature phase called ‘martensite’. The atoms themselves move for much less than interatomic distances, which shears the crystal structure. This makes martensitic transformations a priori much faster than phase transitions that require the exchange of atoms by diffusion. The latter requires much higher temperatures and longer time scales, which limits the switching time, e.g. of phase change memory technology [7]. Martensitic transformations are of first order and accordingly proceed by nucleation and growth, two processes, which occur at the microstructural level. Here we address the question, how fast a complete sample can transform in a fraction of a second, which comprises both underlying processes: the nucleation rate, and the growth velocity of these nuclei.

Steel, a prominent material showing a martensitic transition, has been quenched since historical times to increase its hardness, unwittingly benefitting from the martensitic phase. Nowadays, it is well known that in iron these irreversible transformations can propagate with a velocity of up to one-third of the speed of sound [8]. Further, early experiments revealed a shortest timescale of about 0.1 µs, as summarized by Nishiyama [9]. A similar timescale is observed by shock wave experiments [10]. Here, we focus on reversible martensitic transformations, as switching forth and back between both phases is required for the applications mentioned in the beginning. These so-called thermoelastic transformations can be modelled by molecular dynamics [11,12], which indicates that a martensitic transformation can start and proceed already within several picoseconds. However, these calculations are limited to very short length scales and depend on boundary conditions. Experiments typically focus on the reverse transformation from the martensite back to the austenite during heating. This is because energy can be added much faster into a sample than heat can be removed by dissipation. Fast heating can, for example, be realized by passing a current through a shape memory wire [13]. By Joule heating, pulses as short as a µs have been realized and a subsequent transformation has been observed within 50 … 100 µs. However, for these experiments, the wires had to be prestrained within the martensitic state in order to probe the change of length. The approach is invasive, as inertia limits the response time. Recently, this drawback has been overcome by probing stress instead of strain, which revealed that the transformation within the wires starts after around 20 µs [14]. Simultaneously performed in-situ diffraction experiments showed that the transformation at the wire surface starts as early as 10 µs [15]. In an alternative approach, a magnetic pulse of 13 ms was used to probe magnetic shape memory alloys [16]. Due to their magnetostructural coupling, this allows to induce a phase transformation, which was probed indirectly by a change of magnetization. In this case, dynamics are limited by the long pulse duration. Much shorter pulses can be realized by lasers, but this approach has only been applied to iron, which does not exhibit thermoelastic transformations. When a laser pulse was used to heat an iron film while recording microstructural changes by ultrafast electron microscopy, the bcc-fcc transformation occurred within 220 ns [17]. Finally, lasers with ultra-high power can be used to induce shock waves, and the combination with dedicated in-situ diffraction experiments revealed that the stress induced bcc-hcp transformation in iron can occur within 2 … 4 ns [18]. In general, experiments are sparse, especially for thermoelastic transformations, as it is challenging to induce the phase transformation on short timescales and measure it non-invasively.

To probe how fast we can drive a thermoelastic martensitic transformation, we selected a dedicated setup (more details are given in the experimental section and supplementary Fig. S1). For heating, we used a 7 ns short laser pulse and chose a thin film sample with a thickness of 500 nm. Heat diffusion results in a temperature profile with acceptable homogeneity for this thickness on the timescale of the excitation pulse. As martensitic material, we selected Ni-Mn-Ga, a prototype for Heusler alloys, which exhibit excellent actuation [3,19], sensing [20] and caloric [5] properties. We used synchrotron X-ray diffraction to probe the structural transformation since this method measures the whole film thickness. The synchrotron provides X-ray pulses with a duration of 100 ps, which allows to measure dynamics in very short timescales. Moreover, a variable delay time between laser pulse and X-ray pulse makes our setup ideally suited to probe the transformation in situ in the ns range. To obtain sufficient intensity for these pump-probe experiments, the films were grown epitaxially [21], which ensures that the diffracted intensity is concentrated in just a few peaks. In the examined film, the martensite to austenite transformation starts at around 336 K and is finished at around 365 K as obtained from diffraction measurements (see supplementary Fig. S2 for details). To investigate the speed of the phase transformation and the amount of energy required, we varied the initial sample temperature and heating rate.

## 2. Materials and methods

The investigated, epitaxial sample was prepared by magnetron-sputter deposition in a UHV chamber (base pressure of around 2 × 10^−9^ mbar) from a Ni_44_Mn_32_Ga_24_-target. During deposition, the used MgO(0 0 1) substrate was heated to 400°C and rotated to ensure a homogeneous composition. For better adhesion of the thin film, a 20 nm Cr-buffer layer was deposited underneath the 500 nm Ni_50_Mn_30_Ga_20_ film. The composition was measured with energy-dispersive X-ray spectroscopy (EDX) using an XFlash Detector on a JEOL JSM-6510 (Japan) scanning electron microscope (SEM) with an accuracy of about 1 at.%. An SEM micrograph of the sample is shown as supplementary Figure S6.

For time-dependent diffraction measurements, the beamline P23 at DESY (storage ring PETRA III) was used. The beam energy was chosen to be 12.7 keV, which corresponds to a wavelength of 0.98 Å. To induce the martensite to austenite transition of the sample, the film was heated up with a nano-second laser (Ekspla NL202 (Vilnius, Lithuania), pulse duration 7 ns (full width at half maximum, FWHM), wavelength = 1064 nm, repetition rate = 1 kHz). Though the laser light is absorbed by the electrons, on this timescale the electron and phonon systems are in equilibrium. The base temperature of the sample could be adjusted with a heater from Anton Paar (Model DHS 1100, Graz, Austria (Headquarters)). The laser energy was measured with a power meter (PM100D, Thorlabs, Newton NJ, USA (Headquarters)) for the different laser fluences used in the experiment. The laser spot of approximately 612 × 589 μm^2^ exceeds the X-ray beam footprint of around 10 µm^2^. Furthermore, the spot is much larger than the film thickness. Thus, we obtained structural information of a homogeneously heated film fraction during the heating and subsequent cooling. The diffracted beam was detected with an X-spectrum Lambda 750 K (Germany) detector, which has a spatial resolution of 55 µm and was positioned at a distance of around 0.34 m to the sample. A delay generator (Stanford Research DG645, USA) ensured that the detector only summed up the intensity at particular times, which were selected depending on the different measurement routines. For the data points, the detected intensity for each time interval originated from a single bunch of electrons with an X-ray pulse duration of 100 ps. Due to some inherent characteristics of the laser, the delay at which the pulse is initiated changes depending on the used laser fluence. Accordingly, the time *t* = 0 was defined based on the gathered data points. It was set as the time when the intensity of the involved phases starts to show rapid changes due to the fast heating. All the other points in time are specified with respect to this zero-point.

## 3. Results

**Figure 1.**
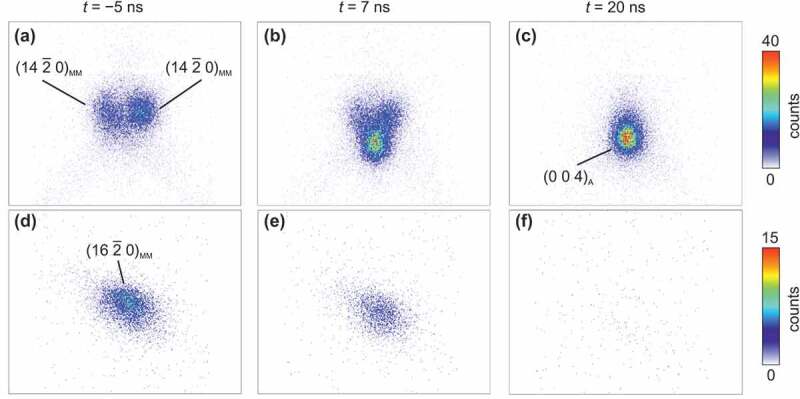
Detector images taken while heating with a laser pulse reveal the time-dependent transformation from martensite to austenite. The images in the first column are taken in the martensite state, 5 ns before the pulse (a, d); those in the second column at the end of the pulse at 7 ns, where both phases coexist (b, e); and those in the third column after the more or less complete transformation of the sample region at t = 20 ns (c, f). Both rows show the raw data in detector pixel coordinates. The pictures in the first row (a–c) show the area around the (0 0 4)_A_ and neighboring ($14\overset{ˉ}{2}0$)_MM_ reflections; the second row shows the area around ($16\overset{ˉ}{2}0$)_MM_ , where no overlap with the austenite occurs (d–f). These exemplary diffractograms have been taken at an initial sample temperature of T_0_=312 K while heating with a laser pulse of 60 mJ cm^−2^, which results in a temperature increase of ΔT*=177K. A full time series of a larger region around (004)_A_ is available as a supplementary video. Indexing follows [20] where the index A denotes an austenite reflection and MM one of modulated martensite. The intensity in all images is scaled linearly according to the scale given on the right side for both rows.

Diffraction is a non-invasive method to investigate a martensitic transformation by directly probing the changes in the crystalline structure. As an example, the diffractograms in Figure 1 illustrate that heating with a 7-ns-long laser pulse, which impinges the material at *t* = 0 ns, can be sufficient to fully transform the sample from martensite to austenite within 20 ns. Due to the required time resolution, the intensity in these diffractograms is low, despite examining a relatively thick film. In the limited time of our synchrotron experiment, we could only position our detector at two small key regions, which were selected following a recent crystallographic analysis of epitaxial films [22]. In the first region (Figure 1(a–c)), the vicinity of (0 0 4)_A_ austenite and ($14\overset{ˉ}{2}\;0$)_MM_ modulated martensite peaks allows to observe both phases in a single detector image. In (A), the sample is martensitic before the laser pulse and is fully transformed to the austenite after around 20 ns in (C). At the end of the laser pulse at *t* = 7 ns (Figure 1(b)), peaks of both phases are visible simultaneously and are separated, as expected for a first-order phase transition. For the second region, the ($16\overset{ˉ}{2}0$)_MM_ peak of the martensite was selected, which is well isolated from all other peaks (Figure 1(d–f)). The nearly complete transformation to the austenite is visible here as well, as the martensite peak disappears at *t* = 20 ns (Figure 1(f)). For the following more detailed analysis of the time dependency, we used the summed-up intensity of this peak.

To understand the influence of fast heating, one first has to convert the applied laser fluence to a temperature rise ΔT* with respect to the base temperature *T*_0_ of the sample. As described in more detail within the supplementary (Figure S3), we use the high brilliance of synchrotron radiation, which allows for a calibration of temperature through the thermal expansion of the austenite lattice [23,24]. We confirmed the validity of this conversion of laser fluence into ΔT* by numerically calculating the temperature evolution from the thermal and optical properties of Ni-Mn-Ga (see supplementary Figure S3). For the martensite to austenite transformation, the overheating Δ*T* is the most important quantity, which we define as the temperature increase above the austenite start temperature *T*_AS_: Δ*T* = *T*_0_+ΔT*-T_AS_.

**Figure 2.**
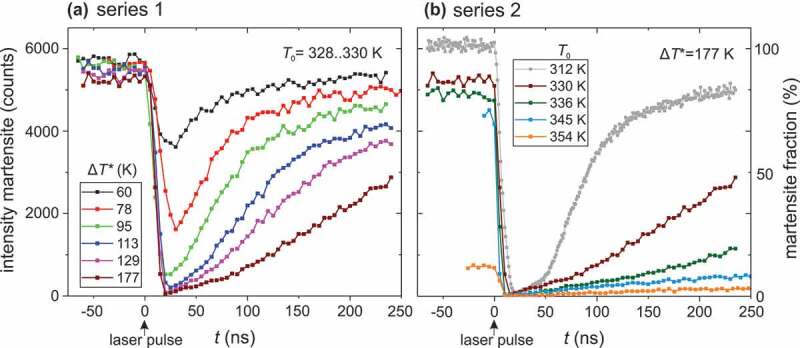
Probing the time dependency of a martensitic transformation while heating with a laser pulse and subsequent cooling. The summed up intensity of the ($16\bar{2}0$)_MM_ peak allows to determine the martensite fraction, which reduces sharply during heating with the 7 ns laser pulse and increases afterwards when the sample cools down. Two series with different experimental conditions were investigated: (a) Nearly the same base temperature of around T_0_ = 329 K was used for all measurements and the laser fluence was varied to obtain temperature rises ΔT* between 60 K and 177 K. (b) the laser fluence and therefore ΔT* was kept constant at 177 K, but the base temperature was varied between 312 K and 354 K.

The influence of fast heating on the martensitic transformation is examined in two experimental series. In the first one, we varied the temperature rise ΔT*. To vary ΔT* between 60 and 177 K, we increased the fluence of the laser pulse from 23 mJ cm^−2^ to 60 mJ cm^−2^ and recorded the diffraction patterns for different delay times. For each measurement, the base temperature *T*_0_ was kept constant at around 329 K, which is just 7 K below the austenite start temperature and 36 K below the austenite finish temperature (see supplementary Figure S2). Exemplary diffraction patterns around the ($16\overset{ˉ}{2}0$)_MM_ peak before, during, and after the laser pulse are depicted in Figure 1(d–f)). For each time step, we summed up the peak intensity, which is proportional to the martensite fraction. From this, we directly obtain the time evolution of the martensite fraction (Figure 2(a)), which proceeds similarly for all ΔT* examined. Before the laser pulse (*t* < 0 ns), the martensite intensity and thus fraction are high. As soon as the laser pulse hits the sample, the martensite fraction drops strongly within a few ns because the sample is heated and transforms into austenite. After the laser pulse, the martensite fraction increases again on a much longer timescale due to cooling. We first analyse the time interval where the amount of martensite phase decreases due to the laser heating and cover the time afterwards, when cooling takes place, in the next paragraph. When using a low laser fluence, which results in a relatively low ΔT* of 60 K, the martensite intensity decreases by about 30% after the laser pulse. With increasing fluence, the fraction of martensite is reduced further, and the transformation proceeds faster. When using the maximum laser fluence (equal to ΔT*  = 177 K), the intensity of the martensite peak disappears almost completely within the duration of the laser pulse. Thus, for large overheating, the short time of 7 ns is sufficient for the complete martensite to austenite transformation. One question remains, which we address in the following: why does a full transformation require 177 K of heating using a short pulse when around 30 K between austenite start and finish is sufficient in quasistatic experiments?

After the laser pulse, the heat of the thin film is dissipated mostly by heat conduction through the substrate and marginally by radiation loss at the surface. During this cooling process, the sample transforms from the austenite to the martensite state. Therefore, the measured intensity increases (Figure 2(a)). The transformation back to martensite proceeds much slower than the martensite to austenite transformation. The process is slowest for the maximum temperature rise of 177 K, where after the first 250 ns only about half of the sample volume has transformed back into the martensite. A complete backward transition can require up to several hundred µs, when the base temperature lies within the transition region. Indeed, for the maximum laser fluence, the 1 ms spacing of the laser pulses is insufficient for the sample to fully cool down to the original base temperature between the laser pulses. This leads to a slight increase in the base temperature from 328 K to 330 K. Thus, *T*_0_ approaches the transformation temperature, which is why for *t* < 0 and ΔT*  = 177 K the intensity is slightly reduced compared to the curves with lower ΔT*, as evident in Figure 2(a). It is worth to add that some heat dissipation already occurs during the short heating by the laser pulse [25]. However, our approach to calibrate the temperature rise by diffraction inherently accounts for this. In summary, the slower dynamics for the transformation during cooling are controlled by heat dissipation, which hinders probing the intrinsic timescale of the austenite to martensite transition. As we aim to understand the fundamental limit, our detailed analysis focuses on the transformation during heating.

In a second series, we varied the base temperature of the sample *T*_0_, while using a constant laser fluence equivalent to ΔT* = 177 K (Figure 2(b)). With increasing *T*_0_, the sample approaches the austenite finish temperature and accordingly, we already start with a smaller martensite fraction before the laser pulse. When heated with high laser fluence, the sample transforms fast and almost completely for all base temperatures. Afterwards, during cooling, the martensite fraction approaches the initial state again. However, this process is much slower at an increased base temperature. We attribute this to the reduced temperature difference between base temperature and martensite finish temperature, which is driven by the heat dissipation during the end of the transformation. This effect is most pronounced for *T*_0_ = 354 K, which according to the arguments in the last paragraph also exhibits the lowest base intensity. This hampered the detailed analysis described later, and thus this measurement was excluded together with the measurement at *T*_0_ = 345 K, where we failed to record enough data points for *t* < 0. Nevertheless, the high surface-to-volume ratio of our thin film leads to an accelerated heat exchange compared to bulk. For a sufficiently low base temperature of 312 K, at least 80% of the austenite transforms back to the martensite within the first 200 ns – much faster than any report before for a ferroelastic transformation from austenite to martensite.

**Figure 3.**
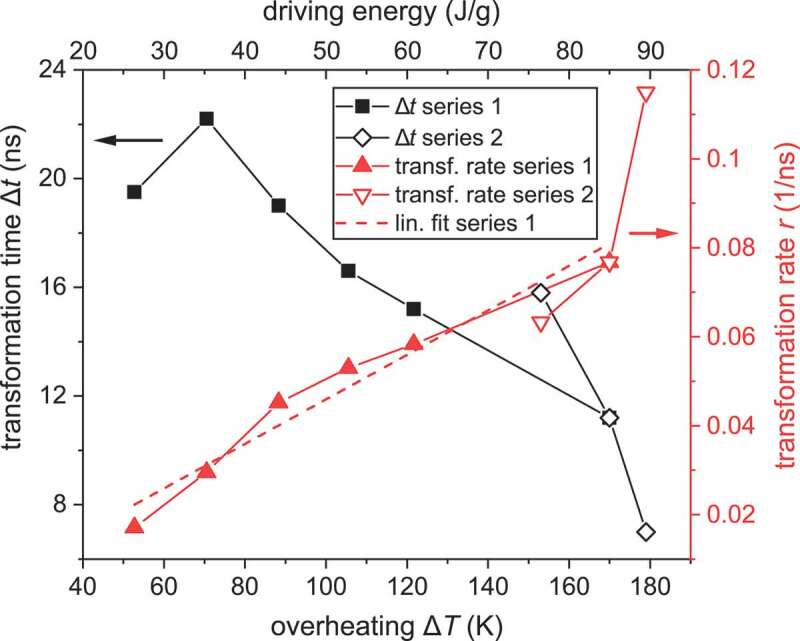
Driving a martensite to austenite transformation as fast as possible. To complete the transformation in a short time interval Δ*t*, a sufficient overheating Δ*T* above the austenite start temperature by the 7 ns laser pulse is required (bottom axis). The laser pulse drives the transformation by adding thermal energy to the sample, which is plotted as additional top axis. An increase of driving energy increases the transformation rate *r* (right axis). This graph contains data obtained for series 1 (solid symbols) and series 2 (open symbols); the latter exhibits a larger experimental error, as described in the text. Therefore, we use only data from series 1 for the linear fit.

In this paragraph, we describe how we convert the experimental results to quantitative values that describe how fast one can drive a martensite to austenite transformation. For this, we take the amount of overheating Δ*T* (as defined above) as a control parameter since this can be directly converted to the driving energy needed for the transformation. In Figure 3 the driving energy is used as the top axis, which is the added thermal gravimetric energy density obtained by multiplying Δ*T* with the mean heat capacity of 500 J kg^−1^ K^−1^ for Ni-Mn-Ga [26,27]. The characteristic values of the transformation, the transition time ∆*t* and transformation rate *r*, were obtained by fitting the martensite fraction with a logistic function and are plotted in Figure 3 for the fluence and temperature series. We define ∆*t* as the time interval in which 90% of the intensity change occurs (see supplementary Fig. S5 for a detailed description). The transformation rate *r* = (sample fraction transformed)/Δ*t* uses the sample fraction transformed, which is the intensity change in relation to the fully martensitic state. The transformation rate exhibits some scatter (cf. Figure 3), which originates from the low intensity typical for cutting edge time resolved experiments. Both series exhibit the same trend, but the slope of series 2 differs, which we attribute to the reduced intensity in the vicinity of the transformation temperature, as discussed in the previous paragraph. This is also the case for the first data point in series 1 at Δ*T*  = 55 K, where only 30% of the sample transforms during the laser pulse. Nevertheless, Figure 3 clearly reveals that increasing overheating reduces the transition time (black curve). The origin of this speed limit is analyzed by looking at the red curve, which reveals the increase in transformation rate with driving energy. We used a linear fit on the transformation rate for series 1, which entails two parameters with scientific impact: First, the threshold overheating of about 8 K, which is found by extrapolating the linear fit to *r* = 0, and second, the driving energy accelerating the transformation, which corresponds to the slope of 10^6^ g(Js)^−1^ for the linear fit.

First, we analyze the threshold overheating Δ*T* of about 8 K, required for a thermally accelerated transformation rate. Above this threshold, we observe a linear relation between transformation rate and driving energy, which is expected from a general thermodynamic viewpoint, as it represents an Onsager relation [28]. Below this threshold, for martensitic transformations peculiarities are observed, which violate this relation [29–31]. In so-called athermal martensite, the transformation only depends on temperature, but not on time. This rate-independent behaviour originates from the complex energy landscape exhibiting shallow local minima along the transformation path, which results in a discontinuous transformation, proceeding by avalanches during nucleation and growth [32]. Indeed, often an asymmetry between both transformation directions is observed [33,34], and thus microscopic reversibility as one key precondition for an Onsager relation is violated during common, low overheating. However, for the huge overheating used in our experiments, the shallow minima become irrelevant and only the major energy barrier between both phases is important. Accordingly, a martensitic transformation follows a different path when completed at a short timescale. This dynamic reversibility explains why we observe an Onsager relation even at a timescale below 10 ns. Moreover, our observation of an Onsager relation indicates a local thermodynamic equilibrium within this timescale, as this is another prerequisite for this relation. As a transition between athermal and thermally accelerated transformation, we propose to use the heating rate, which in our experiments is about 10^9^ K/s at threshold Δ*T*. For applications, we suggest remaining within the athermal regime, as this avoids the additional energy required to accelerate the transformation. We would like to add that in our experiments we could only vary the heating rate by laser fluence but not by pulse duration. Thus, we propose further experiments around the transition region in order to understand the transition between athermal and thermal behavior in more detail.

Second, beyond the athermal regime we measure a slope of the transformation rate of 10^6^ g(Js)^−1^, which describes how much energy is required to accelerate the transformation. To put this value into perspective, we scale it to one Ni_2_MnGa unit cell of 242 u, which gives 2.5 × 10^27^ (Js)^−1^. The unit cell is the fundamental entity to distinguish different thermodynamic states of the material. In a crude approximation, a tetragonal martensitic unit cell has three states, as it can point to any of the three possible orthogonal directions. This bit of information is deleted when the sample is heated to cubic austenite. Considering this as a distinct and complete set of thermodynamic states enables a comparison with the Margolus – Levitin theorem [35], which states that the fundamental speed limit for switching one bit of information is 3 × 10^33^ (Js)^−1^. Indeed, this theorem states quite general: ‘The average energy *E* (…) of an isolated physical system tells us directly the maximum number of mutually orthogonal states that the system can pass through per unit of time’. [35] Computation – and martensitic transformations – are thus just two particular examples for this fundamental speed limit.

In this paragraph, we discuss the limitations and accuracy of our experiments. In order to enable these experiments, we had to make a compromise on film thickness. On one side, we need a thick film to maximize the diffracted intensity from our film. On the other hand, the laser light is absorbed within just several tens of nanometers, which results in an inhomogeneous temperature profile over the film thickness. As a compromise, we selected a film thickness of 0.5 µm, with a diffraction efficiency of 75% of the incident x-rays, and calculated the temperature evolution during and after the laser pulse (supplementary Fig. S3). Taking the values for the maximum laser fluence of 60 mJcm^−2^ as an extreme case, we obtain an average temperature of 600°C at the end of the laser pulse, whereas the top of the film reaches 820°C and the bottom is just at 430°C. This temperature distribution limits the accuracy of our analysis. As the temperature at the surface is about 35% higher than average, our measurements may overestimate the slope of the transformation rate by this percentage in the pessimistic case that we mostly probe only the hotter surface. On the other hand, we also probe the colder, bottom part, which transforms slower. A homogeneous temperature profile should therefore result in a faster transformation than our measurements. From the temperature difference between the average and bottom, we thus expect an underestimation of the transformation time by about 30%. A further limitation in our experiment originates from the thermal stress due to the temperature difference between the hot film and the cold substrate. As worked out in detail in supplementary section 6, this stress-induced martensite can increase the transition temperature between 9 and 53 K when heating up the film by the maximum ΔT* = 177 K. The large variation originates from the unknown initial stress state and accordingly we cannot correct this contribution to the driving energy of the martensitic transformation. Thus, our measurements underestimate the driving energy by 30%. To wrap up, probing the speed limit of martensitic transformation is an experimental challenge. Though our measurements of the slope of the transformation rate can have an error up to exceeding 50%, they give a first idea about the fundamental speed limit of martensitic transformations at all. To improve the experimental accuracy by future synchrotrons with higher brilliance, we propose to use thinner films as they exhibit a more homogeneous temperature profile. To eliminate the stress between film and substrate, freestanding films might be used, but their handling is difficult when they are thin.

Though our integral measurements only reveal how fast the transformation of a whole sample can occur, for future experiments it is interesting to consider also the underlying microstructural processes, which at common time scales consist of nucleation and growth, as described within the introduction. Coexistence of both phases is a prerequisite for both processes, and our experiments indicate that this is also the case at this timescale (Figure 1). However, the observed coexistence may also originate from the inhomogeneous temperature profile, as discussed in the last paragraph. Furthermore, the temperature inhomogeneity can have an impact on the nucleation process. In particular, some martensite may have remained in the colder part of the film, which can act as nuclei and thus accelerate the transition. At the huge overheating used in our experiments, however, nucleation may not be the limiting process anyway since even the classical homogeneous nucleation model predicts an exponential increase in nuclei. In case that many nuclei are present, they do not need a high growth velocity to transform the whole sample. An SEM micrograph of the present film (Supplementary Figure S6) reveals that already after slow cooling a finely twinned microstructure is present. More fundamentally, our observation of a thermally accelerated transformation in contrast to athermal transformations at common timescales indicates for a fundamentally different transformation path, as discussed above. Accordingly, we expect substantial changes in the martensitic microstructure after fast cooling. We propose to use dedicated methods with a high spatial and temporal resolution [17] to probe microstructural changes in detail. We speculate that the decisive microstructure consists of some 10^6^ unit cells, which could explain the difference between the speed limit we observe and the ultimate speed limit by the Margolus – Levitin theorem, which assumes that each unit cell can switch independently.

## 4. Conclusions

To conclude, our dedicated setup allows probing the speed limit of a martensite to austenite transformation. This transformation can be completed below 10 ns, but requires an overheating of several hundreds of Kelvin. For most applications, only a much smaller overheating can be reasonably applied, but also then the switching time is still in the sub-microsecond scale. The austenite to martensite transformation, although limited by heat dissipation in our experiments, can be completed at least within hundreds of ns. This leaves plenty of room to increase the performance of all applications based on martensitic transformations. Our analysis further reveals that driving a martensite to austenite transformation is limited by the product of energy and switching time of around 2.5 × 10^27^ (Js)^−1^, which approaches the ultimate limit determined by the Margolus – Levitin theorem. Thus, this speed limit has the same origin as in microelectronics, where today’s computers just reach 10^10^ (Js)^−1^ – a value which has increased exponentially over the years following Koomey’s law [36].

Despite the fundamental speed limit, the overall performance of microelectronic devices still increases due to the ongoing miniaturization. This path is also accessible for martensitic microsystems since the required total energy is proportional to the volume. Furthermore, the reduced size accelerates heat dissipation, which is decisive for a fast martensite to austenite transformation. Thus, the ultimate performance of martensitic microsystems may just be limited by finite size effects – a topic not yet examined at all.

## Supplementary Material

Supplemental Material

Supplemental Material

## Data Availability

Measured synchrotron data and calculated thermal evaluation during irradiation with the laser pulse are available at https://rodare.hzdr.de/ DOI: 10.14278/rodare.1465. Supplementary information is available at (https://doi.org/10.1080/14686996.2022.2128870), which contains more details on the sample used, calculated temperature profiles, and data analysis [37–49].
