# Supplementary material for: What is the speed limit of martensitic transformations?
Source: Sci Technol Adv Mater. 2022 Oct 6;23(1):633–41. doi: 10.1080/14686996.2022.2128870 (PMC9542621; doi:10.1080/14686996.2022.2128870)
Supplement: Supplemental Material [file TSTA_A_2128870_SM9201.pdf]

Supporting Information

What is the speed limit of martensitic transformations?

*Stefan Schwabe, Klara Lünser, Daniel Schmid, Kornelius Nielsch, Peter Gaal, Sebastian Fähler**

* s.faehler@hzdr.de

1) Set-up for pulsed laser heating and time resolved synchrotron diffraction

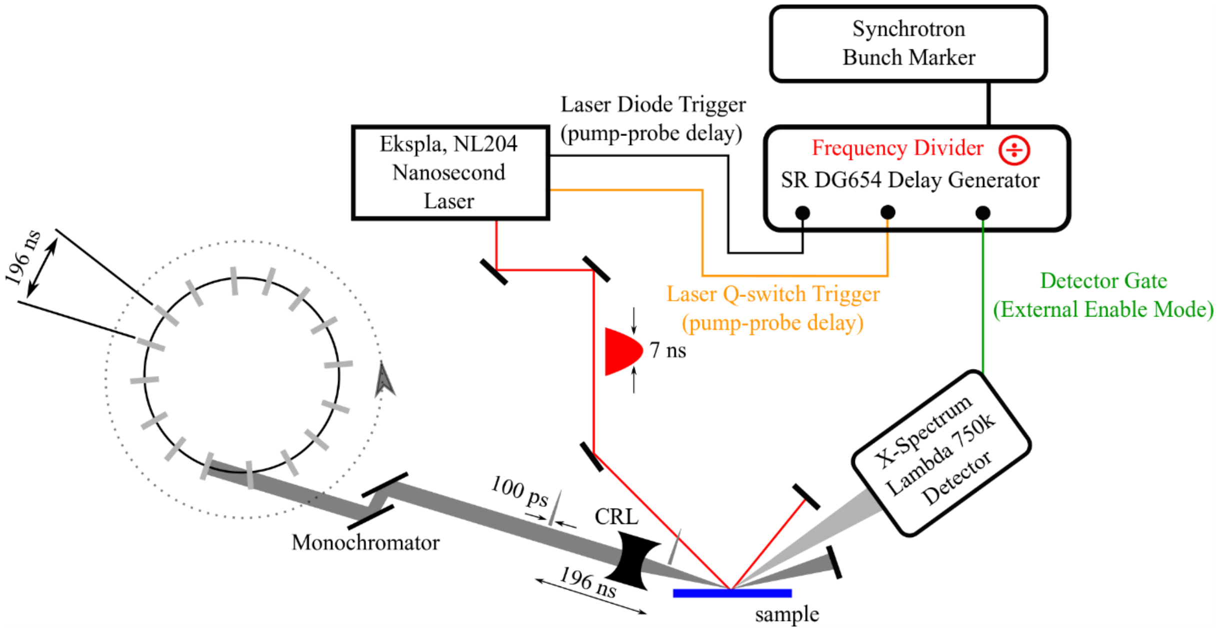

Fig. S1: Sketch of the experimental setup. A q-switched laser (Ekspla, NL204) is synchronized to the Petra III bunch marker using a Stanford Research DG654 Delay Generator and delivers optical pulses at a wavelength of 1064 nm with a pulse energy and a pulse duration of up to 2 mJ and 7 ns, respectively. 100 ps X-ray pulses from the Petra III storage ring are monochromatized and focused onto the sample to probe transient structural dynamics in the sample. The diffracted signal is captured by a Lambda 750k area detector in external gating mode. The gating reduces the acquisition rate to 1 kHz, i.e., the same rate as the optical excitation pulses. This sketch was adapted from [37].

2) Estimated radiation transmission rate

To estimate if the synchrotron radiation probes the complete film thickness, we calculated the ratio of transmitted X-ray for our film.

$$\frac{I_1}{I_0} = e^{-\mu x}$$

I_0 and I_1 are the X-ray intensities before and after the film, μ is the linear attenuation coefficient of the material and x the distance travelled in the film. To give information about the whole film, the radiation has to travel through the film to the substrate and back out again. As the radiation hits the film in an angle of 18° and with a film thickness of 500 nm, x equals 3200 nm. With μ of 887 cm^{-1} (for Ni_2MnGa with a density of 8 g cm^{-3} and a beam energy of 12.1 keV, [38]), $\frac{I_1}{I_0}$ calculates to 75 %, which means that still a reasonable amount of the x-ray radiation is not absorbed in the film. Therefore, we can assume that the synchrotron radiation gives information from the complete film thickness.

3) Characterization of the temperature dependent phase transition

To select reasonable base temperatures for our pump-probe experiments, a thorough understanding of the transition temperatures of the material is necessary. Therefore, the phase transition of the investigated Ni-Mn-Ga thin film was studied using magnetization as well as resistivity measurements, in addition to the diffraction experiments. Fig. S2 (a) shows the temperature-dependent magnetization of the sample, which was measured by a vibrating sample magnetometer (VSM) in a PPMS system (VersaLab™, 2 K/min heating and cooling rate in an external magnetic field of 0.01 T). The phase transition shows a hysteresis and is accompanied by a change in magnetization at around 350 K. As the transition partly overlaps with the Curie-temperature at 366 K, an additional resistivity measurement was performed in another PPMS system. The resulting curve in zero field is shown in Fig. S2 (b) in black and for clarity only the reverse transition is plotted. As the austenite has a lower resistivity than the martensite, there is a clear drop at the phase transition. For comparison, the phase fraction of the martensite was also measured at the beamline using the diffracted intensities of the $(16\ {-2}\ 0)_{MM}$ martensite peak without laser heating. The red curve in Fig. S2 (b) shows this phase fraction for particular temperatures, which were held steady within ±0.3 K for the measurement time. To obtain the martensite phase fraction, the intensity was normalized to the maximum intensity at 306 K. Both curves show a similar transition region, which is shifted to

slightly lower temperatures for the curve derived from the diffraction experiments. This may be attributed to the different measurement setups, as the thermometers are placed at different distances to the sample. Furthermore, during the resistivity measurement, the temperature was swept with a rate of 3 K/min compared to the quasi-static diffraction experiments. To determine the austenite start and finish temperatures, we thus used the curve obtained by X-ray diffraction as the corresponding temperature measurement setup was the same one used for all time-dependent measurements at the synchrotron. As with the time dependent measurements, we defined the austenite start temperature as the point were 5 % of the sample has transformed and the finish temperature for 95 % transformation, which gives 336 K and 365 K, respectively. An interesting point that can only be seen from the diffraction experiment (red curve) is that even at 400 K – considerably above the sharp intensity drop – the transition to the austenite is not fully completed yet. This aspect is only visible in the direct diffraction experiments and not by the commonly used indirect methods like magnetization and resistivity.

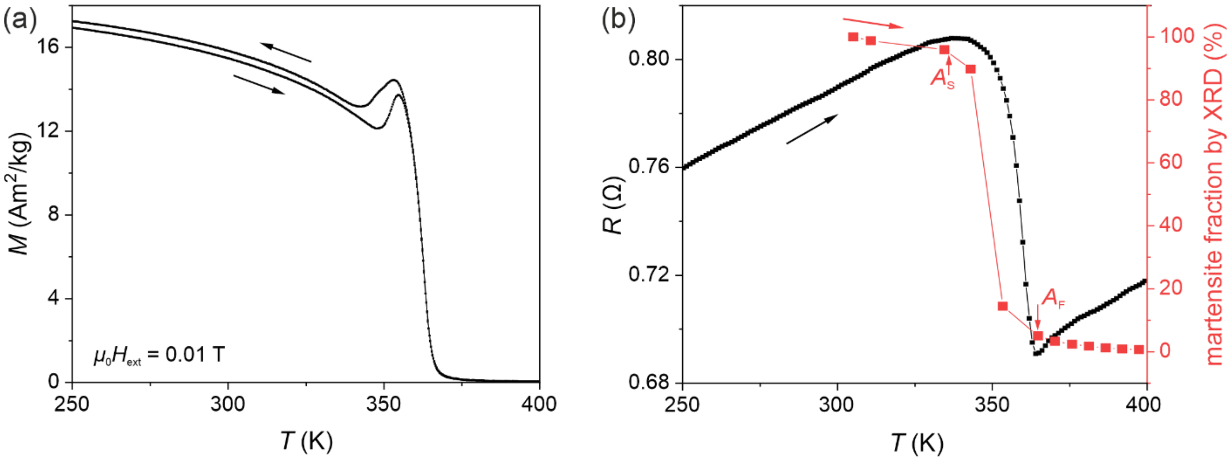

Fig. S2: Determining transition temperatures in quasi-static measurements.
(a) Temperature dependent magnetization of the investigated sample measured with VSM in a PPMS system (VersaLab™). An external field of 0.01 T was applied. As the phase transition partially overlaps with the Curie-temperature (T_c = 366 K), an additional resistivity measurement was performed in another PPMS system in zero field ((b), black curve). For comparison, the temperature-dependent intensity of the martensite phase measured quasi-statically at the beamline is shown in red in vicinity of the transformation temperature. Both curves in (b) only depict the reverse transformation. All measurements reveal a similar transformation behavior and slight differences in temperature can be attributed to the different setup and sweep rates. Therefore, we used the values obtained directly at the beamline: austenite start A_S=

336 K at 95 % martensite fraction and austenite finish A_F= 365 K at 5 %, as marked in the graph.

4) Determination of the laser induced temperature rise

For the detailed evaluation of the results gathered from the time-dependent diffraction experiments, it is necessary to probe the temperature rise ΔT induced by the laser. To obtain this, we use the high accuracy of diffraction to probe lattice parameters, which allows to measure the thermal expansion. Thus, the lattice constant of the Ni-Mn-Ga film itself is the “thermometer”, and accordingly ΔT is representative of the relevant sample region and thickness. The calculation of the X-ray radiation transmitted for the present film (section 2) of the supplementary) reveals a diffraction efficiency as high as 75 %, and accordingly we consider these measurements a good average over the film thickness. For these measurements we heated the sample to 413 K into the fully austenite state to avoid any possible variant reorientation within the martensitic state. We chose the maximum laser fluence of around 60 mJ cm^{-2} and monitored the position of the austenite (004) peak perpendicular to the substrate. From that, we derived the change in lattice parameter (Δa_0) in relation to the initial value before the laser pulse hit the sample, which is plotted in Fig. S3.

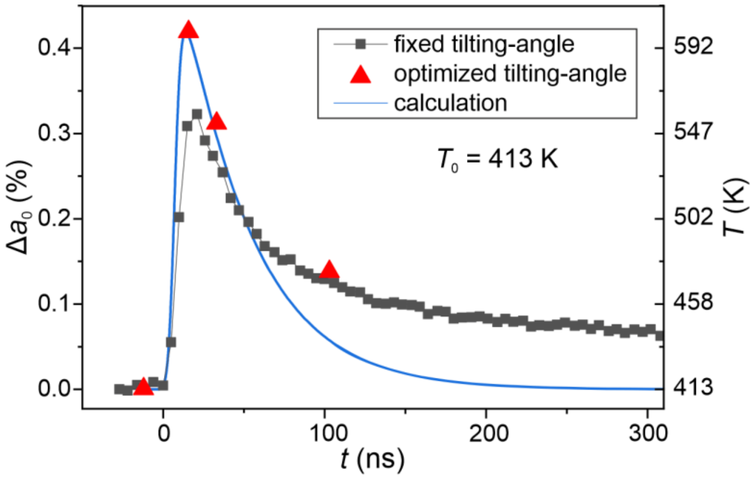

Figure S3: Determining the laser induced temperature rise. Time-dependent change of lattice parameter of the austenite (a_0) measured while heating the sample with a laser fluence of 60 mJ cm^{-2} from a starting temperature of 413 K. Respective temperatures obtained from a calibration at quasistatic temperatures are shown on the right y-axis. The gray data points were collected with constant diffractometer tilt angles. The red data points show some discrete values with diffractometer angles adjusted for the

maximum intensity of the $(004)_A$ austenite peak at selected points in time, as described in detail within the text. The blue line shows the calculated temperature development.

The lattice parameter of the film increases sharply while the sample is heated up and decreases afterwards while the sample "slowly" cools down. The black points were recorded continuously with a constant set of diffractometer angles. However, the fixed detector and change of lattice parameter can mean that one does not perfectly hit the austenite reflection in reciprocal space, which may result in an apparent slight variation in lattice parameter. To exclude this possible error, additional experiments where performed, where for some selected times the diffractometer angles were optimized for intensity. These measurements are plotted in red and show some deviations from the fixed diffractometer measurements, especially in the time interval directly after the laser pulse hit the sample. Therefore, the red data point with the maximal change of lattice parameter is used further on.

To calibrate our "Ni-Mn-Ga thermometer", we measured the thermal expansion coefficient in out of plane direction for our sample in a laboratory diffractometer and obtained a value of 22.4×10^{-6} K^{-1}. Though we used the same sample for calibration, the accuracy of this approach is limited by the different temperature profile and thermal expansion (see section 7 of this supplementary), since during the calibration the complete substrate is hot, whereas during pump probe experiments most of the thick substrate remains at ambient (see section 5 of the supplementary). This calibration allows us to convert the maximum change in lattice parameter into a temperature change, which results in $\Delta T^* = 187$ K for a laser fluence of 60 mJ cm^{-2}. The conversion for all the other laser fluence values used in our investigation was done assuming a linear relationship between laser fluence and temperature change. To account for the latent heat of the transformation, we compared the latent heat to the specific heat capacity of Ni-Mn-Ga. With a latent heat of around 5000 J kg^{-1} [39,40], and a specific heat capacity of around 500 J kg^{-1} K^{-1} [26,27], around 10 K of the ΔT will be absorbed by the material as latent heat. We compared the measured values with calculations using the thermal and optical properties of Ni-Mn-Ga (blue line in Figure S3). The maximum temperature change of $\Delta T^* = 187$ K is confirmed by the calculation, described in detail in the next section. However, according to the calculation, the temperature decreases faster after the laser

pulse than in the experiment. We attribute this to the film-substrate interface that acts as a heat transfer coefficient, which is not taken into account in the calculation.

5) Calculation of temperature profile during and after laser pulse

To calculate the depth dependent temperature of film and substrate we use the python-based toolbox *udkm1Dsim* [41,42]. This toolbox uses a simplified molecular dynamics model to calculate one-dimensional propagation of coherent and incoherent acoustic phonons. A number of previous measurements showed that at these short laser pulses and large spot sizes heating and subsequent cooling occurs exclusively in the direction perpendicular to the sample surface, i.e., a one-dimensional modelling is sufficient [23]. The simulated structure consists of 1000 unit cells of Ni-Mn-Ga (585 nm) and 30000 unit cells of MgO substrate (12.63 µm). The large thickness of the substrate allows employing a constant temperature boundary condition at the rear side of the substrate. At the sample surface we employ a thermal isolation boundary condition. The complex index of refraction for Ni_2MnGa was taken from literature [43] and slightly adapted to match the observed peak temperature rise in the Ni-Mn-Ga film after optical excitation. The value employed in the simulations was 2.0+1.32j. Thermal properties of bulk Ni-Mn-Ga and MgO were taken from [43] and [44], respectively. The optical excitation pulse is absorbed in the upper 65 nm Ni-Mn-Ga film. At this pulse duration, heat within the film dissipates already during absorption of the laser pulse into the substrate. Thus, the generated peak temperature is approximately 10 times lower compared to the excitation with a ultrashort (sub 1 ps) optical pulse. Figure S4 (a) displays the temperature as a function of time and film depth obtained from our simulations. The temperature at the middle of the film matches well the mean film temperature (see Figure S4 (b)).

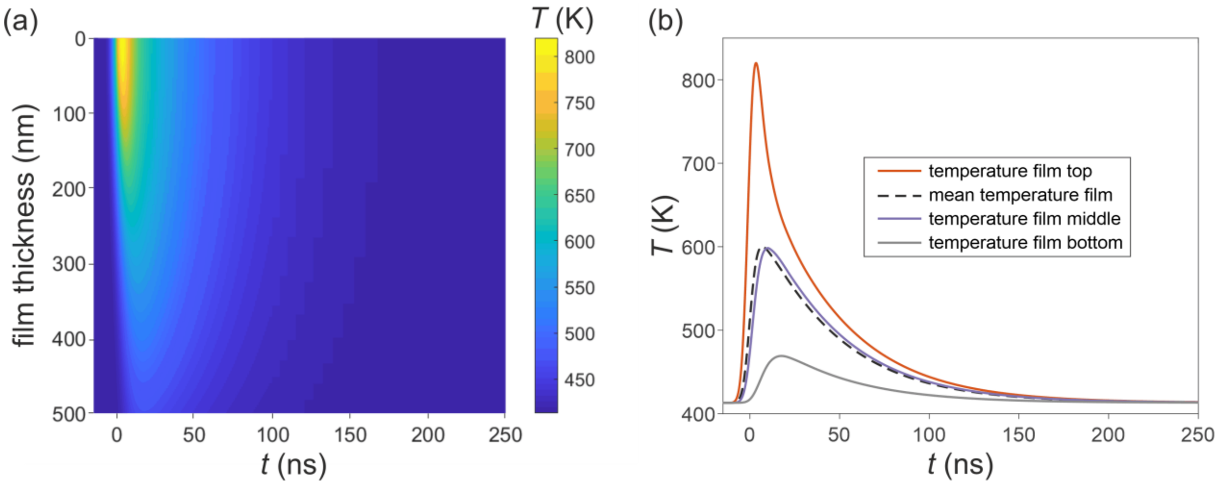

Fig. S4: Calculated temperature profile of the Ni-Mn-Ga film on MgO substrate during and after the laser pulse. The temperature as a function of the film depth and the time was calculated with finite elements. The calculations assumed a start temperature of 413 K and a laser fluence of 60 mJ cm^{-2} (a) The heat map shows the evolution of the temperature throughout the film after the laser pulse at t = 0 ns. (b) Temperature profile at specific depths of the film (top, middle and bottom side). The dashed curve displays the mean temperature of the complete film.

6) Obtaining characteristic values from the time-dependent intensity measurements

As described in the main part of the paper, our thin film sample was investigated for various different laser fluences as well as base temperatures. To compare the time-dependent phase transition for these different experimental conditions, we first had to extract some characteristic parameters from the intensity data. Starting with the impact of the laser pulse, the intensity curves for the martensite to austenite transition are Z-shaped as more and more of the sample transforms until a maximum of transformed phase is reached. To derive characteristic values from these curves, we fitted the part of the curve in which the intensity decreases using a generalized logistic function. This function can be used to describe saturation and transition processes and requires only a small number of fitting parameters. Three exemplary curves with the corresponding fit (red) are shown in Figure S5 (a-c). The generalized logistic function to describe the time dependency of intensity $I(t)$ has theform:

$$I\,(t) = \mathrm{UB} - \frac{\mathrm{UB} - \mathrm{LB}}{\left(1 + e^{-b\cdot(t-t_0)}\right)^{\frac{1}{v}}}$$

UB refers to the upper boundary of the function, i.e., the maximum it approaches. LB is the lower boundary, t_0 shifts the position along the time axis, and the parameters *b* and *v* describe the shape of the drop. *b* is the transition rate and *v* affects how the asymptote is approached. After fitting this function to our data, we derived the following key parameters of a transition process: while UB and LB can be taken directly from the fit, we are additionally interested in the transition time Δt, which is sketched in blue in Figure S5 (a). We define Δt as time span required for the fit function to decrease from 95 % to 5 % of its maximum drop (UB − LB). This approach gives the transition time, which is the key parameter to understand the speed of a martensitic transformation. In particular, this approach allows neglecting details of the curve shape, which may be affected e, g. by the inhomogeneous temperature profile, described in supplementary section 6). We use the generalized logistic function, as it directly give UB and LB and a slightly better fit to our data compared to the standard one. Together with Δt these data could also be extracted directly from our measurements by the classical tangent method, but with lower accuracy. For completeness, all fit parameters are summarized in the following table.

Table S1: Fit parameters of the generalized logistic function on Series 1 and 2. ΔT^* and T_0 in the first two rows give the parameters used for the respective measurements. For ΔT^* of 177 K and T_0 of 336 K, we did not measure enough data points to make a fit possible.

ΔT^* (K)	60	78	95	113	129	177	177	177	177	177
T_0 (K)	330	330	330	330	330	330	312	336	345	354
UB	5735	5618	5604	5502	5390	5230	6081	-	4893	717
LB	3711	1629	383	156	0	0	0	-	0	0
t_0 (ns)	6.3	7.9	2.8	9.9	4.5	4.8	9.6	-	3.5	6.9
b (1/ns)	0.3	0.2	0.2	0.3	0.3	0.4	0.2	-	0.9	0.8
v	0.7	0.3	0.3	0.7	0.2	0.2	0.02	-	1.2	1.4
Δt (ns)	20	22	19	17	15	11	16		7	9

A generalized logistic function can describe the data points quite well. For the measurement shown in (a), we used a small time increment describing the intensity drop very detailed. Due to the limited measurement time at a synchrotron, we had to increase the step size for most of the experiments as can be seen in (b) and (c). The fit is still able to reproduce the data, which is also the case when the sample only partially transforms as

shown in Figure S5 (c). Depending on the experimental conditions, the transition time differs noticeable from well below 10 ns in (b) to nearly 20 ns in (c).

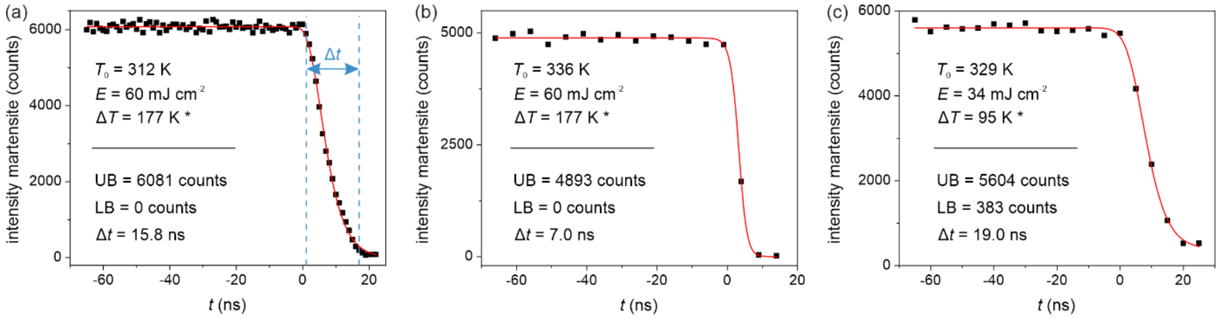

Fig. S5: Analyzing time-dependent measurements while heating the sample with a nanosecond laser pulse (exemplary measurements). The intensity of the martensite $(16\ \text{-}2\ 0)_{MM}$ peak (black) was fitted with a generalized logistic function (red), as described in the text. The upper (UB) as well as lower boundary (LB) of the function and the derived transition times (Δt, sketched in blue) are given in the figure. The base temperature before the laser pulse (T_0) was 312 K (a), 336 K (b) and 329 K (c). The laser fluence (E) and the temperature rise ΔT is given in the corresponding graphs as well. As described in supplementary section 4), ΔT was corrected for the latent heat during the transition (*) by subtracting 10 K from the values obtained from the measurements shown in Fig. S3.

7) Estimation of film stress and influence on transformation temperature

In our analysis we focus on the influence of temperature, but the difference of thermal expansion of the hot, thin film on the cold, thick substrate can result in a film stress, which commonly increases the martensitic transformation temperature. Following our analysis of thermal stress in Ni-Mn-Ga films [45] we estimate the increase of transition temperature by thermal stress for the maximum temperature rise of 177 K. From the thermal expansion coefficient of 22.4 x 10^{-6} K^{-1} of Ni-Mn-Ga (see section 4) of this supplementary) we expect a strain of 0,4 %, which is equivalent to a stress of 120 MPa when using the E-module = 20 GPa of austenite [46]. Following the Clausius-Claperyon equation of Ni-Mn-Ga [47,48], this stress increases the transition temperature by 54 K. This, however, is an upper estimate, as this stress is compressive, and films after growth often exhibit tensile stress [45], which can compensate most of the thermal stress. When using the tensile stress of this paper as an approximation (the stress of the present film is not known), the increase of transition temperature is reduced to 9 K. When putting these

values in relation to the temperature rise of 177 K, we underestimate the driving energy by 5 % to 30 % by neglecting thermal stress.

We would like to add, that at no time the surface temperature of our film (Fig. S4a) approaches the melting point and thus no shock wave by evaporating forms, as used in other dedicated setups [49].

8) Microstructure of the examined Ni-Mn-Ga film

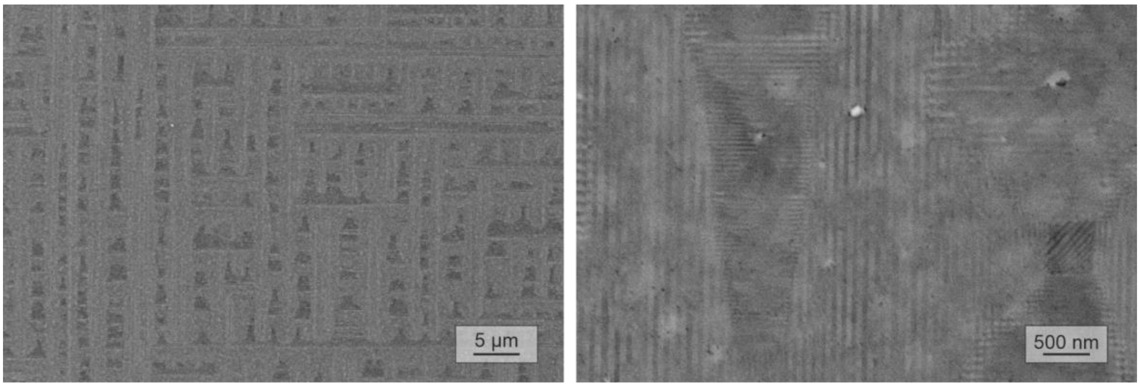

Fig. S6: SEM micrographs of the examined Ni-Mn-Ga film in different magnification. The martensitic microstructure consists mostly of Type Y and the mesoscopic twin boundaries are 20…50 nm apart. We would like to add that we recently published a comprehensive explanation how and why this hierarchical microstructure forms (after slow cooling) [22].

[22] Schwabe S, Niemann R, Backen A, et al. Building Hierarchical Martensite. Adv Funct Mater. 2021;31:2005715.

[23] Shayduk R, Navirian H, Leitenberger W, et al. Nanoscale heat transport studied by high-resolution time-resolved x-ray diffraction. New J Phys. 2011;13:093032.

[26] Devarajan U, Kannan M, Thiyagarajan R, et al. Coupled magnetostructural transition in Ni-Mn-V-Ga Heusler alloys and its effect on the magnetocaloric and transport properties. J Phys Appl Phys. 2015;49:065001.

[27] Porcari G, Cugini F, Fabbrici S, et al. Convergence of direct and indirect methods in the magnetocaloric study of first order transformations: The case of Ni-Co-Mn-Ga Heusler alloys. Phys Rev B. 2012;86:104432.

[37] Schmidt D, Bauer R, Chung S, et al. A new concept for temporal gating of synchrotron X-ray pulses. J Synchrotron Radiat. 2021;28:375–382.

[38] Chantler CT. Detailed Tabulation of Atomic Form Factors, Photoelectric Absorption and Scattering Cross Section, and Mass Attenuation Coefficients in the Vicinity of Absorption Edges in the Soft X-Ray (Z=30–36, Z=60–89, E=0.1

keV–10 keV), Addressing Convergence Issues of Earlier Work. J Phys Chem Ref Data. 2000;29:597–1056.

[39] Caballero-Flores R, Sánchez-Alarcos V, Recarte V, et al. Latent heat contribution to the direct magnetocaloric effect in Ni–Mn–Ga shape memory alloys with coupled martensitic and magnetic transformations. J Phys Appl Phys. 2016;49:205004.

[40] Polyakov PI, Slyusarev VV, Kokorin VV, et al. Volume Change During Intermartensitic Transformations in Ni-Mn-Ga Alloy. J Mater Eng Perform. 2014;23:3180–3183.

[41] Schick D. udkm1Dsim – a Python toolbox for simulating 1D ultrafast dynamics in condensed matter. Comput Phys Commun. 2021;266:108031.

[42] Schick D, Bojahr A, Herzog M, et al. udkm1Dsim—A simulation toolkit for 1D ultrafast dynamics in condensed matter. Comput Phys Commun. 2014;185:651–660.

[43] Kuo YK, Sivakumar KM, Chen HC, et al. Anomalous thermal properties of the Heusler alloy $Ni_{2+x}Mn_{1-x}Ga$ near the martensitic transition. Phys Rev B. 2005;72:054116.

[44] Slack GA. Thermal Conductivity of MgO, Al_2O_3, $MgAl_2O_4$, and Fe_3O_4 Crystals from 3° to 300°K. Phys Rev. 1962;126:427–441.

[45] Thomas M, Heczko O, Buschbeck J, et al. Stress induced martensite in epitaxial Ni–Mn–Ga films deposited on MgO(001). Appl Phys Lett. 2008;92:192515.

[46] Chernenko VA, Seguí C, Cesari E, et al. Sequence of martensitic transformations in Ni-Mn-Ga alloys. Phys Rev B. 1998;57:2659–2662.

[47] Chernenko VA, Pons J, Cesari E, et al. Stress–temperature phase diagram of a ferromagnetic Ni–Mn–Ga shape memory alloy. Acta Mater. 2005;53:5071–5077.

[48] Chernenko VA, L'vov V, Pons J, et al. Superelasticity in high-temperature Ni–Mn–Ga alloys. J Appl Phys. 2003;93:2394–2399.

[49] Meyers MA, Gregori F, Kad BK, et al. Laser-induced shock compression of monocrystalline copper: characterization and analysis. Acta Mater. 2003;51:1211–1228.
